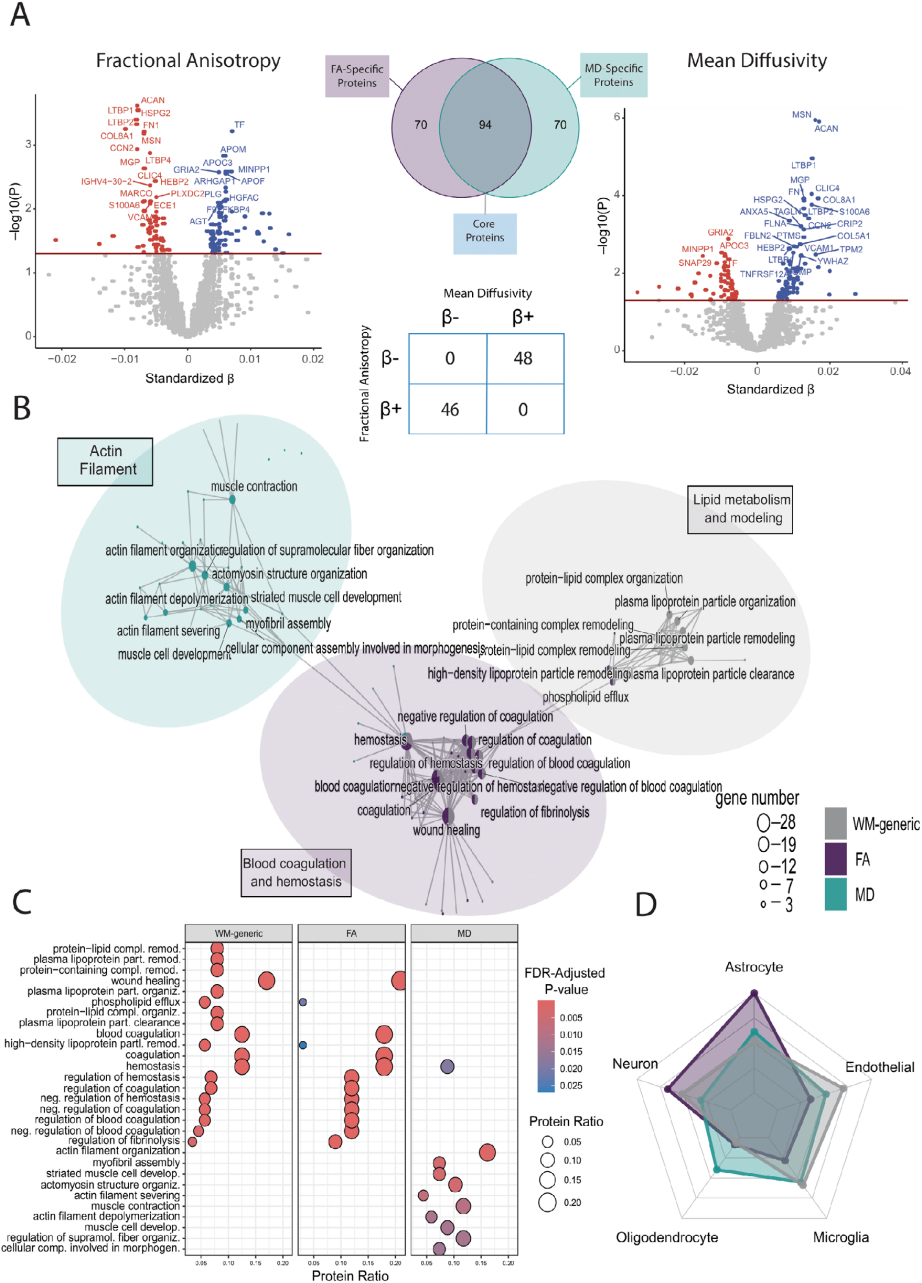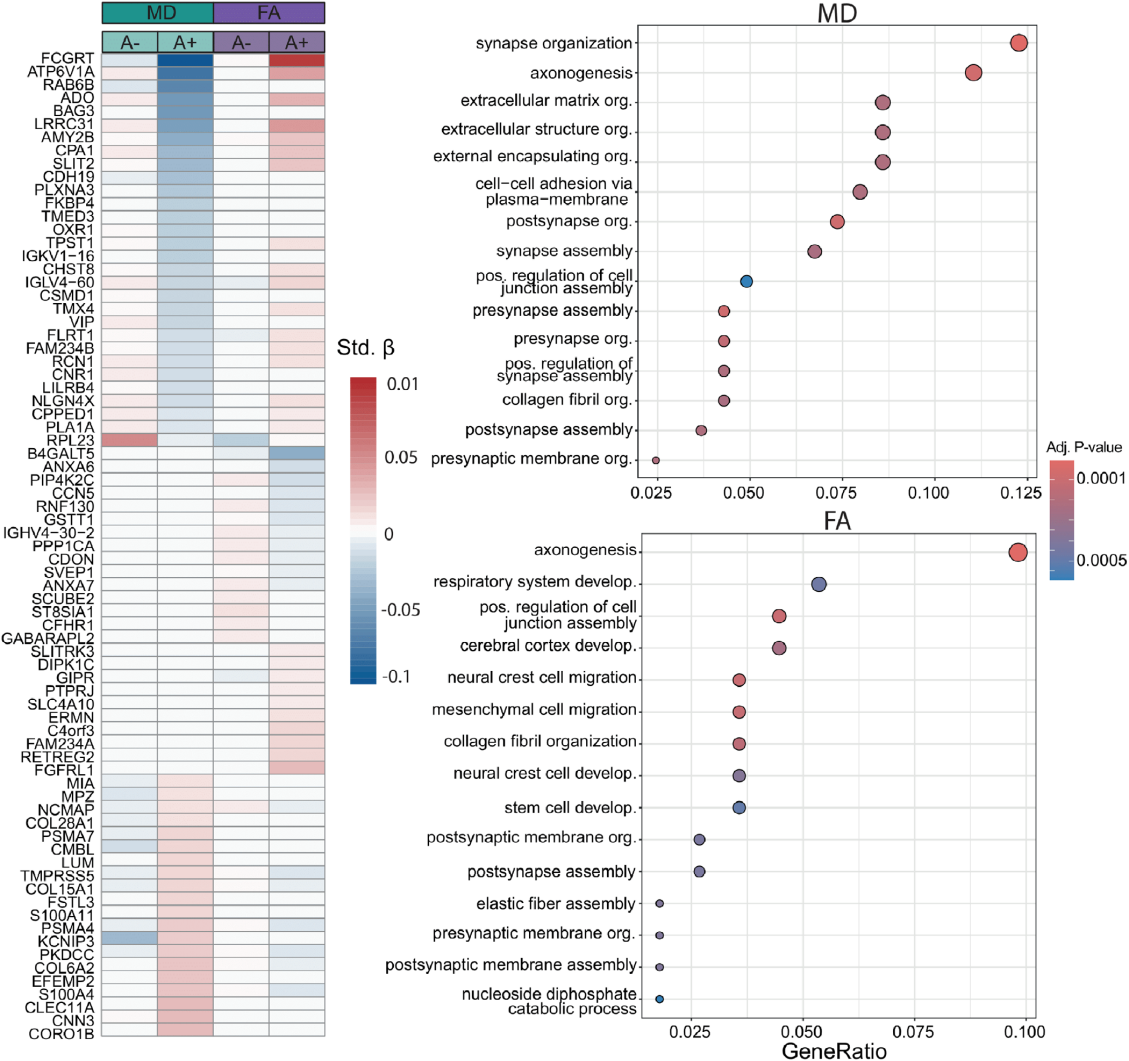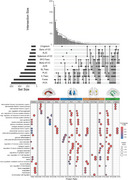# White matter integrity measures relate to distinct CSF proteomic profiles in non‐demented subjects dependent on amyloid status

**DOI:** 10.1002/alz70856_098829

**Published:** 2025-12-24

**Authors:** Luigi Lorenzini, Mario Tranfa, Mara ten Kate, Diederick Martijn de Leeuw, Anouk den Braber, Luca Roccatagliata, Matteo Pardini, Charlotte E. Teunissen, Pieter Jelle Visser, Frederik Barkhof, Betty M. Tijms

**Affiliations:** ^1^ Department of Radiology and Nuclear Medicine, Amsterdam, Amsterdam, Netherlands; ^2^ Amsterdam University Medical Center (Amsterdam UMC), Amsterdam, North Holland, Netherlands; ^3^ Department of Neurology, Alzheimer Center Amsterdam, Amsterdam Neuroscience, Vrije Universiteit Amsterdam, Amsterdam, Netherlands; ^4^ Alzheimer Center Amsterdam, Neurology, Vrije Universiteit Amsterdam, Amsterdam UMC location VUmc, Amsterdam, Netherlands; ^5^ Department of Neuroscience, Ophthalmology and Genetics University of Genoa, Genoa, Italy; ^6^ IRCCS Ospedale Policlinico San Martino, Genova, Italy; ^7^ Neurochemistry Laboratory, Amsterdam Neuroscience, Program Neurodegeneration, Amsterdam UMC, Vrije Universiteit Amsterdam, Amsterdam, Noord‐Holland, Netherlands; ^8^ Alzheimer Center and Department of Neurology, Amsterdam Neuroscience Campus, VU University Medical Center, Amsterdam, North Holland, Netherlands; ^9^ Department of Radiology and Nuclear Medicine, Vrije Universiteit Amsterdam, Amsterdam University Medical Center, location VUmc, Amsterdam, Netherlands; ^10^ Alzheimer Center Amsterdam, Neurology, Vrije Universiteit Amsterdam, Amsterdam UMC location VUmc, Amsterdam, North Holland, Netherlands

## Abstract

**Background:**

Growing evidence indicates a potential role of white matter damage in the onset and progression of Alzheimer's disease (AD). Understanding the biological processes underlying *in‐vivo* white matter imaging biomarkers is essential for broadening their use beyond research settings and identifying new potential targets for disease prevention and modification strategies.

**Methods:**

We selected 96 non‐demented older individuals from the Alzheimer Centrum Amsterdam with available DTI and CSF proteomic data. Fractional anisotropy (FA) and mean diffusivity (MD) values were computed for the total white matter, and for 12 tracts of interest, using tract‐based spatial statistics. We tested associations between protein levels (predictors) and both global FA and MD values (outcomes) with linear models, correcting for age and sex. Models further included an interaction between protein levels and amyloid status. Gene‐set and cell‐type enrichment analysis were performed on proteins showing significant associations.

**Results:**

CSF levels of 234 (17.1%) proteins were associated with global DWI measures (Figure 1). Of these, 70 were unique for FA, and 70 for MD, while the remaining proteins were associated with both measures (WM‐generic proteins). WM‐generic proteins were related to pathways of lipid metabolism and enriched in endothelial cells. FA‐specific proteins were mostly related to blood coagulation pathways and enriched in astrocytes and in neurons. MD‐specific proteins were related to actin filaments and mainly expressed in oligodendrocytes. Testing interaction terms of protein levels with amyloid status (Figure 2), revealed both global FA and MD alterations in amyloid positive participants were associated with biological processes of axonogenesis, synaptic organization and plasticity. Regional analysis revealed distinct proteomic profiles associated with variations in regional FA and MD, with processes linked to synaptic plasticity and axon extension specifically related to limbic and posterior projections fibers (Figure 3).

**Conclusions:**

These results provide new insights into the biological mechanisms underlying white matter integrity and its spatial relationship to AD pathology. The observed amyloid‐dependent associations underscore the interplay between amyloid pathology and white matter damage, suggesting that interventions targeting synaptic organization, axonogenesis, and plasticity may hold promise for mitigating disease progression.